# Task- and Intensity-Dependent Modulation of Arm-Trunk Neural Interactions in the Corticospinal Pathway in Humans

**DOI:** 10.1523/ENEURO.0111-21.2021

**Published:** 2021-09-28

**Authors:** Atsushi Sasaki, Naotsugu Kaneko, Yohei Masugi, Tatsuya Kato, Matija Milosevic, Kimitaka Nakazawa

**Affiliations:** 1 Graduate School of Arts and Sciences, Department of Life Sciences, The University of Tokyo, Tokyo 153-8902, Japan; 2 Japan Society for the Promotion of Science, Tokyo 102-0083, Japan; 3 School of Health Sciences, Tokyo International University, Saitama 350-1197, Japan; 4 Graduate School of Engineering Science, Department of Mechanical Science and Bioengineering, Osaka University, Osaka 560-8531, Japan

**Keywords:** arm-trunk interaction, corticospinal pathway, motor evoked potential, transcranial magnetic stimulation

## Abstract

Most human movements require coordinated activation of multiple muscles. Although many studies reported associations between arm, leg, and trunk muscles during functional tasks, their neural interaction mechanisms still remain unclear. Therefore, the aim of our study was to investigate arm-trunk or arm-leg neural interactions in the corticospinal tract during different arm muscle contractions. Specifically, we examined corticospinal excitability of the erector spinae (ES; trunk extensor), rectus abdominis (RA; trunk flexor), and tibialis anterior (TA; leg) muscles while participants exerted: (1) wrist flexion and (2) wrist extension isometric contraction at various contraction intensity levels ranging from rest to 50% of maximal voluntary contraction (MVC) effort. Corticospinal excitability was assessed using motor evoked potentials (MEPs) elicited through motor cortex transcranial magnetic stimulation (TMS). Results showed that ES MEPs were facilitated even at low contractions (>5% MVC) during wrist flexion and extension, while stronger contractions (>25% MVC) were required to facilitate RA MEPs. The extent of facilitation of ES MEPs depended on contraction intensity of wrist extension, but not flexion. Moreover, TA MEPs were facilitated at low contractions (>5% MVC) during wrist flexion and extension, but contraction intensity dependence was only shown during stronger wrist extension contractions (>25% MVC). In conclusion, trunk extensor corticospinal excitability seems to depend on the task and the intensity of arm contraction, while this is not true for trunk flexor and leg muscles. Our study therefore demonstrated task- and intensity-dependent neural interactions of arm-trunk connections, which may underlie anatomic and/or functional substrates of these muscle pairs.

## Significance Statement

Although it is known that most human movements require coordinated activation of multiple muscles, understanding of how they are controlled in the central nervous system still lacks. Our study investigated the characteristics of neural interactions of arm-trunk and arm-leg muscles in the corticospinal tract of human participants using motor evoked potentials (MEPs) elicited by transcranial magnetic stimulation (TMS). We showed that arm muscle contractions can facilitate corticospinal excitability of the trunk and leg muscles. Specifically, arm-trunk neural interactions depended on the task and intensity of arm movements. Our findings therefore suggest that corticospinal neurons have complex output patterns to distinct muscles in different body segments, which may depend on the anatomic and/or functional relationship of these muscle pairs.

## Introduction

Most human movements, even simple acts such as grasping an object, require coordinated activation of multiple muscles. Specially, interactions between arm and trunk muscles are important for performing activities of daily living. It is well known that trunk muscles are activated before the proceeding arm movements (Aruin and Latash, 1995; Hodges and Richardson, 1997). By assessing motor evoked potentials (MEP) using transcranial magnetic stimulation (TMS) of the primary motor cortex (M1), it was recently demonstrated that voluntary activation of upper-limb muscles can facilitate corticospinal circuits, which are responsible for controlling the trunk muscles (Chiou et al., 2018; Sasaki et al., 2018). Moreover, it was reported that subcortical excitability evaluated by cervicomedullary MEP was not changed by voluntary contraction of arm muscles (Chiou et al., 2018; Sasaki et al., 2020b). These results indicate that trunk corticospinal facilitation induced by arm movement may be mediated in the cortical networks. Therefore, motor control centers of trunk and arm muscles may not be embedded within the central nervous system as separate units. Rather, they seem to interact closely. Although corticospinal remote facilitation may be responsible for controlling arm-trunk coordinated movements (Chiou and Strutton, 2020), its mechanisms are yet to be fully understood.

Corticospinal remote facilitation has been studied extensively between upper-limb and lower-limb muscles (Kawakita et al., 1991; Péréon et al., 1995; Tazoe and Komiyama, 2014) and this phenomenon is known as remote effect or crossed facilitation (Tazoe and Komiyama, 2014). Specifically, contraction of upper-limb or lower-limb muscles is known to facilitate corticospinal excitability of muscles located in different and remote segments of the body (Kawakita et al., 1991; Péréon et al., 1995; Hortobágyi et al., 2003; Tazoe et al., 2007b, 2009; Chiou et al., 2013a,b; Komeilipoor et al., 2017). Such interlimb corticospinal remote facilitation was achieved regardless of whether different tasks (e.g., flexion or extension) was performed (Tazoe et al., 2007b, 2009; Chiou et al., 2013a,b; Tazoe and Komiyama, 2014). Moreover, the extent of corticospinal remote effect facilitation between upper-limb and lower-limb muscles was shown to depend on the voluntary effort level of the contracted muscle (Kawakita et al., 1991; Tazoe et al., 2007b, 2009). Regardless of the neurophysiological characteristics of remote effect facilitation, its functional role in human motor control is still unknown (Tazoe and Komiyama, 2014). Moreover, arm-trunk neural interaction mechanisms in corticospinal tract have not been examined in detail, compared with more widely studied interlimb remote effects. Since trunk muscles are activated in a highly coordinated manner during voluntary arm movements (Aruin and Latash, 1995; Hodges and Richardson, 1997), characteristics of arm-trunk neural interactions may be different from those observed in interlimb remote effect facilitation (Tazoe and Komiyama, 2014). Moreover, since previous studies investigating arm-trunk neural interaction used only one contraction intensity [i.e., either 20% or 30% maximum voluntary contraction (MVC) level; Chiou et al., 2018; Sasaki et al., 2018, 2020b; Chiou and Strutton, 2020], it was not clear how neural interactions between arm and trunk muscles would be modulated during different contraction intensities. Therefore, investigating arm-trunk remote effect facilitation during different exertion levels and tasks may provide new insights about underlying neural interaction mechanisms in the central nervous system. Fundamentally, this could lead to a more comprehensive understanding of the basic principles of human motor control. Moreover, it was recently reported that trunk muscle corticospinal excitability could be facilitated after short-term upper-limb training (Chiou et al., 2020). Arm-trunk corticospinal remote facilitation may also be attributed to quicker anticipatory postural adjustments of the trunk during rapid shoulder flexion in patients with spinal cord injury (Chiou and Strutton, 2020). Therefore, a thorough understanding of the characteristic of arm-trunk corticospinal interactions may also be helpful to develop new rehabilitation interventions for targeting improvements in arm-trunk interactions.

It was reported that trunk flexor [i.e., rectus abdominis (RA)] and extensor [i.e., erector spinae (ES)] muscle activity depends on the direction of the arm movements (e.g., flexion or extension) during various motor tasks (Aruin and Latash, 1995; Hodges et al., 1997). We therefore hypothesized that arm muscle contractions would facilitate corticospinal excitability of the trunk muscles, as recently demonstrated (Chiou et al., 2018; Sasaki et al., 2018). Specifically, our hypothesis was that the extent of arm-trunk corticospinal remote facilitation would depend on the task performed during upper-limb muscle contractions (i.e., flexion or extension), as indicated by studies that examined muscle-level outputs (Aruin and Latash, 1995; Hodges et al., 1997). We also hypothesized that muscle contraction intensity would affect the extent of arm-trunk remote effect facilitation, similar to that of interlimb facilitation (Kawakita et al., 1991; Tazoe et al., 2009). Moreover, we also expected that the abovementioned task- and intensity-dependent arm-trunk corticospinal remote facilitation profiles would change depending on the functional role of the trunk muscles (i.e., RA or ES), as suggested by a previous study that showed differences between RA and ES muscles for muscle-level outputs (Aruin and Latash, 1995). Therefore, the overall objective of our study was to first confirm arm-trunk (and arm-leg) remote effect facilitation. If remote effect facilitation was indeed elicited using our current study paradigm, the second objective was to examine whether task (flexion or extension) and intensity (various exertion levels) of muscle contractions would affect remote effect facilitation. To test our hypotheses, we used TMS to investigate MEPs in the trunk extensor and flexor muscles during wrist flexion and extension tasks at the various contraction intensity levels. Lower limb MEPs were also measured under same experimental conditions to identify whether the observed modulations were specific to arm-trunk interactions, or whether co-activation of any two muscles would produce similar facilitation patterns. Moreover, investigating remote effects of multiple body segments, including limb and trunk muscles at the same time under the same condition, could lead to a better understanding of neural interaction mechanisms of multiple muscles in human motor control.

## Materials and Methods

### Participants

Twelve healthy male volunteers were recruited for this study. The age, weight, and height of the participants were 24.8 ± 1.5 years, 67.2 ± 6.6 kg, and 173.5 ± 5.3 cm (mean ± SD), respectively. All participants were right-handed. None of the participants had any history of neurologic or musculoskeletal impairments. Specifically, for the TMS study, we confirmed that all participants had no metal implants, cardiac pacemaker, history of epilepsy, brain injury, neurosurgery, or psychological disorders, have never had a convulsion or a seizure, and did not regularly take medications such as anti-depressants or other neuromodulatory drugs (Rossi et al., 2011). All participants gave written informed consent in accordance with the Declaration of Helsinki. The experimental procedures were approved by the local institutional ethics committee.

### Experimental procedures

**Figure 1.**
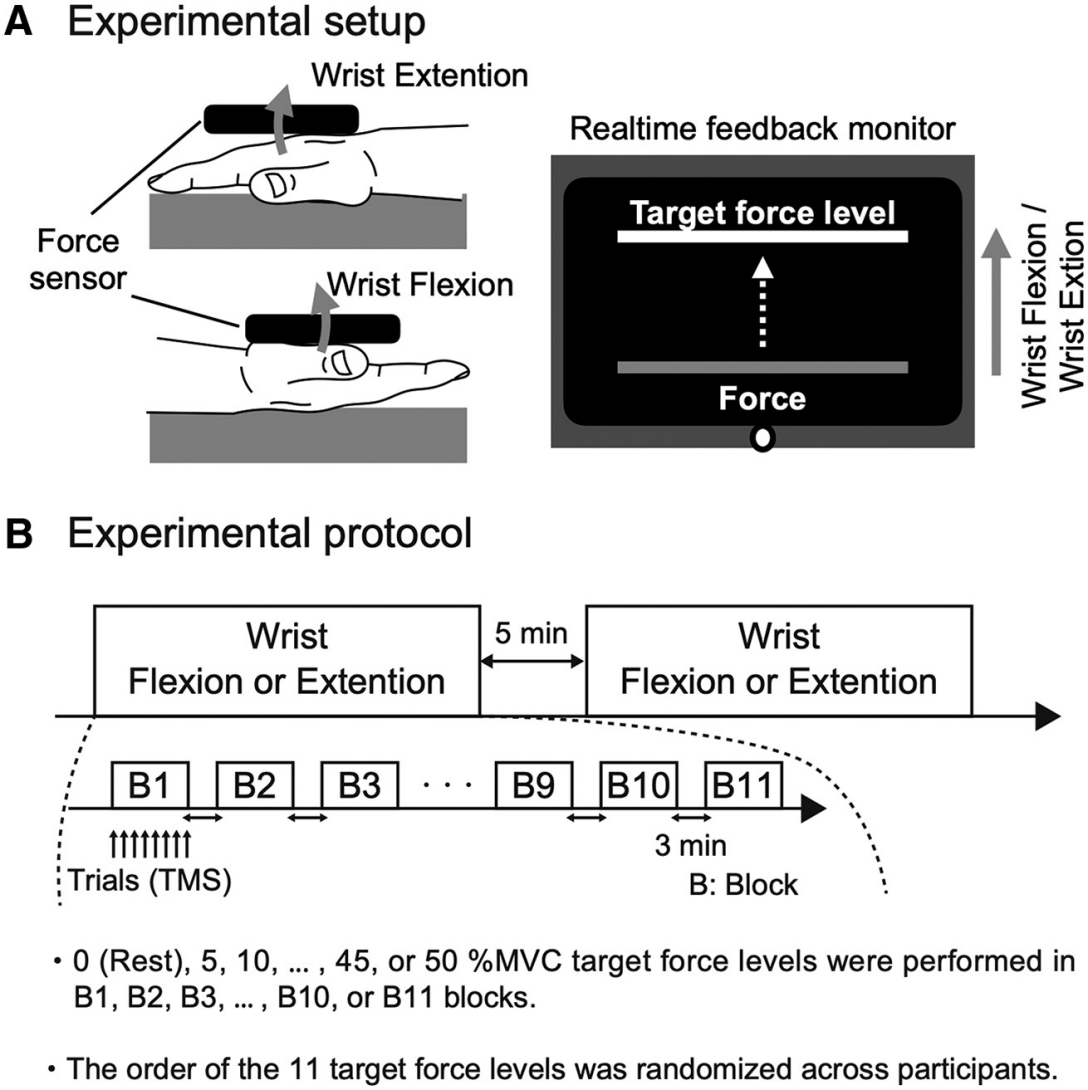
***A***, Experimental setup showing the hand posture of participants during the experiment. During the experiment, participants were asked to match the isometric wrist flexion and wrist extension force 0–50% of MVC effort using their right arm with real-time visual feedback of force displayed on a monitor. ***B***, Experiment consisted of wrist flexion and wrist extension conditions, which were randomized between participants and separated by 5-min rest. Each condition consisted of 11 blocks and each target force [0% (rest), 5%, 10%, …, 45%, or 50% MVC] was randomly set to each block with at least 3-min rest between blocks. Each block consisted of eight trials.

During the experiment, participants were seated comfortably on a chair with a back support to keep their trunk muscles relaxed. Following a gentle warm-up and task practice, MVC level was first measured by asking the participants to perform three isometric wrist flexions and three isometric wrist extensions in a randomized order between participants with their right (dominant) arm. Force level of the wrist flexion and wrist extension was measured using a strain gauge sensor (LCB03K025L, A&D Company Ltd), which was fixed to a metal frame located on the distal part of the forearm (Fig. 1*A*). During the experiments, corticospinal excitability was assessed during: (1) wrist flexion and (2) wrist extension. In order to control for the biomechanical effects of performing flexion and extension movements, both tasks were performed by rotating the arm such that the resultant movements were in the opposite direction to gravity (Fig. 1*A*). During wrist flexion and extension tasks, participants were asked to match the forces corresponding to a range from 0% (rest) to 50% of MVC force level and maintain this contraction intensity by matching the force target level which was displayed on a monitor in real time (Fig. 1*A*). Each task consisted of target force [0% (rest) to 50% MVC with steps of 5%], corresponding to 11 blocks (Fig. 1*B*) which were separated by at least 3-min rest. The order of the target force levels was randomized between participants. TMS stimuli were delivered when participants maintained the corresponding contraction target level for a period of 3–5 s (i.e., steady-state part of the contraction). Each block consisted of eight trials, which were separated with ∼10 s between trials. Moreover, the order of the experimental tasks (flexion or extension) was randomized between participants, with at least 5-min rest between tasks.

### Data acquisition

#### Electromyography (EMG) activity

EMG activities were recorded unilaterally from right side of: (1) ES muscle on the 12th thoracic vertebral level (ES; trunk extensor muscle); (2) RA muscle lateral to the umbilicus (RA; trunk flexor muscle); and (3) tibialis anterior muscle lateral to the tibia (TA; lower-limb muscle). Two bipolar Ag/AgCl surface electrodes (Vitrode F-150S, Nihon Kohden) were placed over the muscle belly with 1 cm separation. A ground electrode was placed over the right anterior superior iliac spine. Before application of electrodes, skin was cleaned using alcohol to reduce impedance. All EMG signals were bandpass filtered (5–1000 Hz) and amplified (1000×) using a multichannel amplifier (MEG-6108, Nihon Kohden). All data were digitized at a sampling frequency of 4000 Hz using an analog-to-digital (A/D) converter (Powerlab/16SP, AD Instruments) and stored on the computer for postprocessing.

#### TMS

TMS was delivered over the M1 using a mono-phasic magnetic stimulator (Magstim 200, Magstim Co) through a double cone coil (outside diameter of 110 mm; Magstim Co). The optimal stimulation spot (“hot spot”) was searched over the left motor cortex where MEPs could be recorded from the right ES muscle. Once the hot spot was defined, the coil position and orientation were monitored throughout the experiment using a neuronavigation system (Brainsight, Rogue Research) to ensure same coil placement between tasks. The motor threshold (MT) was determined while the participants remained relaxed. Specifically, the MT was defined as the minimum TMS intensity for which ES MEPs had peak-to-peak amplitudes larger than 50 μV and were evoked in at least five out of ten consecutive trials (Rossini et al., 2015). The stimulus intensity was set at 120% of the MT level (73.3 ± 12.3% of maximal stimulator output) and remained consistent for the duration of the experiment. Since the highest MT in the current study was 76% of maximal stimulator output, we were able to apply 120% MT level for all participants. During preliminary testing, it was confirmed that we could elicit MEP responses in RA and TA muscles when the stimulation hot spot and intensity were optimized for the ES muscle. However, since two participants for the RA and three participants for the TA had <0.05-mV amplitude of MEPs at rest, these muscles were excluded for MEP analysis (i.e., RA: *n* = 10 and TA: *n* = 9). The average MEP amplitudes with SD at rest in the ES, RA, and TA muscles were 0.08 ± 0.03, 0.34 ± 0.25, and 0.42 ± 0.27 mV, respectively.

### Data analysis

Background EMG activity of a 50 ms window before each TMS stimulus was first defined by calculating the root mean square value in each muscle and each trial using a custom written script in MATLAB (2017a, The MathWorks Inc.). It is well known that MEPs elicited by single plus TMS are facilitated by background activation of the muscle (Hess et al., 1987). Therefore, if trunk and lower-limb muscles were co-activated during upper-limb muscles contraction tasks (i.e., wrist flexion and wrist extension), it would not be possible to evaluate the remote effect. Comparing background EMG activity was therefore used to ensure that remote muscles were not contracted during wrist flexion or extension tasks. If the background EMG activity in any of the experimental tasks was significantly different from rest (0% MVC level), remote effect facilitation was not considered for these tasks (see Results).

To analyze remote effect facilitation, MEP peak-to-peak amplitudes were calculated for each trial and each remote muscle (i.e., ES, RA, and TA). Eight repeated trials were averaged for each task (i.e., wrist flexion and wrist extension) and each contraction intensity (i.e., % MVC contraction levels for which the remote limb muscle background EMG activity was not different from the Rest condition; see Results, Background EMG activity). In preliminary experiments it was determined that eight trials were sufficient to obtain consistent recordings since variability was sufficiently low, consistent with previous studies (Groppa et al., 2012). MEP amplitudes were then normalized as a percentage of the amplitude of the elicited responses during the rest condition for each participant.

### Statistics

**Figure 2.**
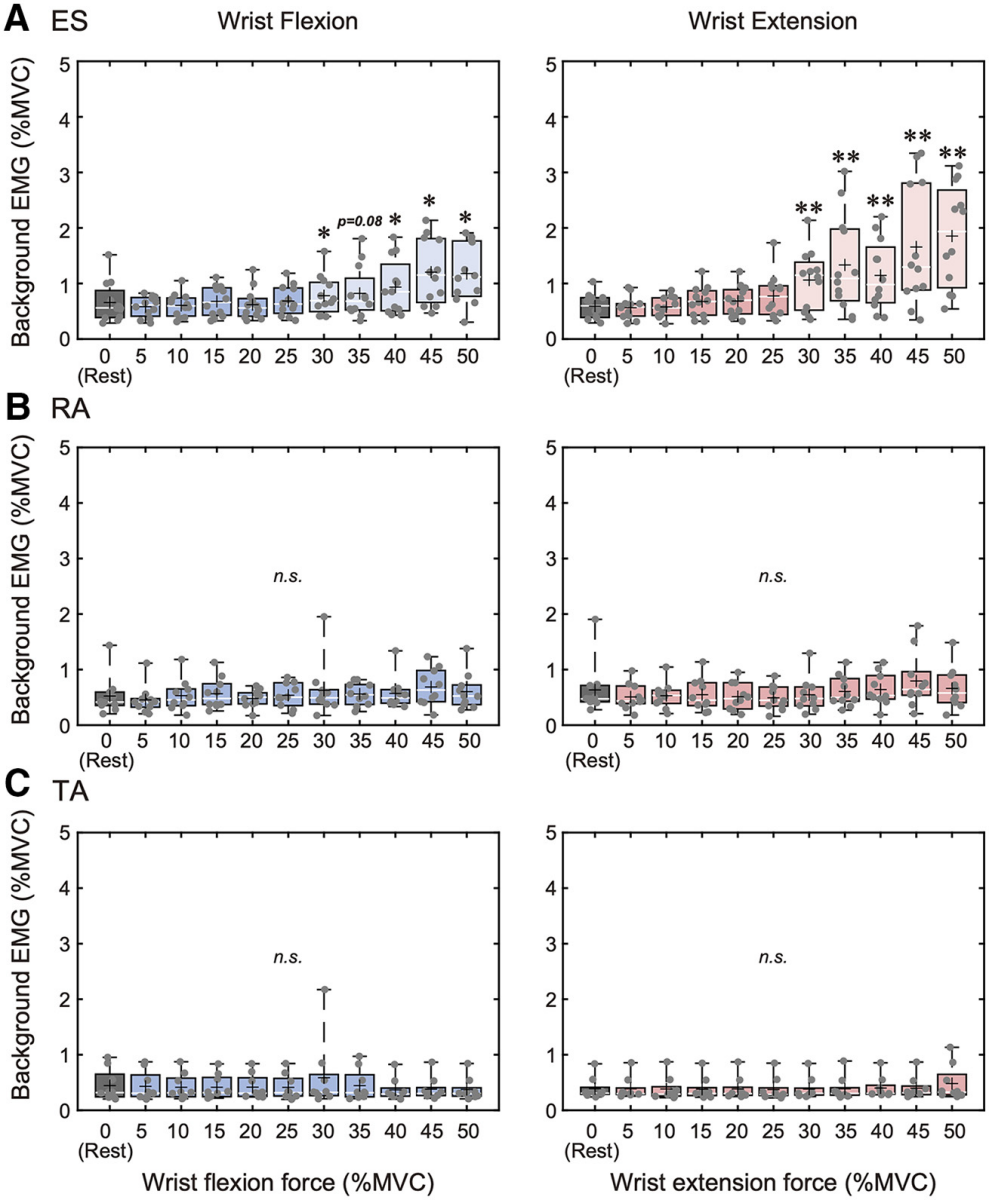
Group data for background EMG activity of the (***A***) ES, (***B***) RA, and (***C***) TA muscles during 0% (rest) to 50% of MVC effort of wrist flexion and wrist extension. The lines and cross marks in the box plots indicate median and mean values, respectively. The ends of the boxes represent the 25th and 75th percentiles. The whiskers on the boxplot illustrate the minimum and maximum values. Asterisks indicate significant differences compared with 0% MVC (rest). n.s., non-significant; *p < 0.05, **p < 0.01.

For each muscle (ES, RA, and TA) and each task (wrist flexion and wrist extension) separately, background EMG activities were first compared between different muscle contraction intensities (rest and 5–50% MVC with steps of 5%) using the Friedman test, a non-parametric equivalent for repeated-measure ANOVA. Significant results were followed up with *post hoc* multiple comparisons using the Wilcoxon signed-rank test to compare rest (0% MVC) to each remote contraction condition level (5%, 10%, …, 45%, and 50% MVC). Since background EMG activities of the ES muscle during wrist flexion and wrist extension at the contraction levels above 25% MVC (i.e., 30%, 35%, 40%, 45%, and 50% MVC) were significantly greater compared with that during rest (0% MVC; Wilcoxon signed-rank test, *p* < 0.05; see Results, Background EMG activity; Fig. 2*A*), only data below 30% MVC (5, 10, 15, 20, and 25% MVC) were used for remote effect MEP analysis for the ES muscle. Background EMG activities of the RA and TA muscle during wrist flexion and wrist extension were not significantly different compared with rest (0% MVC) even if upper-limb muscles were contracted at 50% MVC level (Wilcoxon signed-rank test, *p* < 0.05; see Results, Background EMG activity; Fig. 2*A*). However, initially we also only included data below 30% MVC (5%, 10%, …, 20%, and 25% MVC) for remote effect MEP analysis of the RA and TA muscles since it is possible that their facilitation may also have been affected by the background EMG activity of ES muscles.

For MEP analysis, we first investigated whether remote facilitation occurred in each remote contraction condition (5–25% MVC) using the Friedman test, a non-parametric equivalent for repeated-measure ANOVA. The Friedman test included MEP amplitudes in the rest, 5%, 10%, 15%, 20%, and 25% MVC. Significant results were followed up with multiple comparisons using the Steel *post hoc* test, which is a non-parametric equivalent for Dunnett’s test, to determine whether MEPs during remote contraction conditions (5%, 10%, 15%, 20%, and 25% MVC) were significantly different from Rest for each muscle. When significant remote facilitation was shown, the Friedman test was used to compare MEP amplitudes between contraction at 5%, 10%, 15%, 20%, and 25% MVC to investigate contraction intensity effects on remote facilitation for each muscle. Significant results were followed up with *post hoc* testing using the Wilcoxon signed-rank test with Holm corrections. Specifically, five remote contraction conditions (5%, 10%, 15%, 20%, and 25% MVC) were analyzed in *post hoc* testing, and the statistical significance levels were adjusted using the Holm corrections, as summarized by McLaughlin and Sainani (2014).

Additional analysis was conducted to investigate remote effect contraction intensity dependence in RA and TA muscles during high intensity remote contraction (30%, 35%, 40%, 45%, and 50% MVC) since they were excluded from the main analysis because of larger ES background EMG activities [note: RA and TA background EMG activities were not statistically different compared with rest (0% MVC)]. Specifically, since ES background EMG activities were significantly increased during 30–50% MVC of wrist flexion and wrist extension (see Results, Background EMG activity), it could be considered that activation of ES background EMG (i.e., possible remote effect facilitation from ES to RA and/or TA muscles) may have affected RA and TA corticospinal excitability. Therefore, correlations between the remote effect in RA and TA MEPs and ES background EMG activations during 30–50% MVC of wrist flexion and wrist extension were first analyzed using Spearman’s rank correlations. Since no significant correlations were shown (see Results, MEP modulation during larger contraction intensities), the same statistical tests were conducted for MEP amplitudes between contraction at 30%, 35%, 40%, 45%, and 50% MVC as for those during lower contraction intensities (5–25% MVC).

Overall, non-parametric tests were chosen because the Shapiro–Wilk test showed that most identified measures were not normally distributed. All statistical comparisons were performed using the software package R (version 3.6.3). Significance level for all tests was set to *p* < 0.05.

## Results

### Background EMG activity

The background EMG activity results are shown in Figure 2. The Friedman test showed that ES background EMG activities were significantly different between contraction intensities (0–50% MVC) in both wrist flexion and wrist extension tasks [wrist flexion: χ^2^(10) = 54.9, *p* < 0.001; wrist extension: χ^2^(10) = 82.2, *p* < 0.001]. Specifically, *post hoc* analysis showed that the ES background EMG activities during ≥30% MVC wrist flexion and wrist extension were significantly increased, compared with rest (0% MVC; *p* < 0.05, Wilcoxon signed-rank test; Fig. 2*A*).

The Friedman test showed that RA background EMG activities were not significantly different between contraction intensities (0–50% MVC) during wrist flexion task [χ^2^(10) = 18.2, *p* = 0.052], while they were significantly different during wrist extension task [χ^2^(10) = 26.2, *p* < 0.01]. *Post hoc* analysis showed no significant differences in RA background EMG activities during wrist extension between rest (0% MVC) and remote contraction condition (*p* > 0.05, Wilcoxon signed-rank test; Fig. 2*B*).

Finally, the Friedman test showed that TA background EMG activities were not significantly different between contraction intensities (0–50% MVC) in both wrist flexion and wrist extension tasks [wrist flexion: χ^2^(10) = 11.1, *p* = 0.354; wrist extension: χ^2^(10) = 9.19, *p* = 0.514; Fig. 2*C*].

### MEP modulation during low contraction intensities

**Figure 3.**
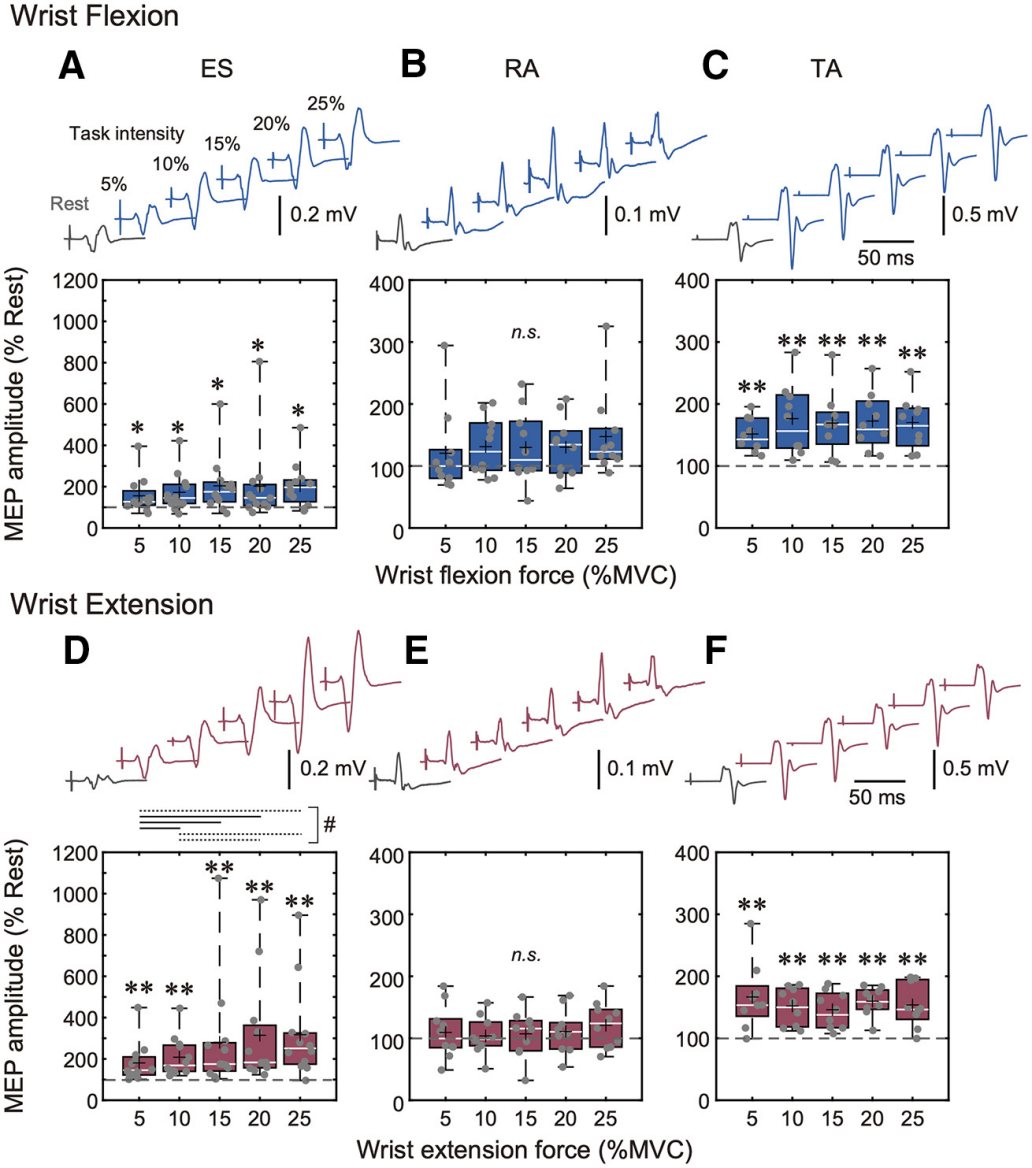
***A*–*C***, Wrist flexion condition: averaged MEPs in the ES, RA, and TA muscles of one representative subject during 0% MVC (rest: gray traces) and 5–25% MVC (remote effect: blue traces). Box plots show group data for MEPs elicited in the ES, RA, and TA muscles. ***D*–*F***, Wrist extension condition: averaged MEPs in the ES, RA, and TA muscles of one representative subject during 0% MVC (rest: gray traces) and 5–25% MVC (remote effect: red traces). Box plots show group data for MEPs elicited in the ES, RA, and TA muscles. All MEP amplitudes were normalized with respect to the MEP amplitude at 0% MVC (rest) for each participant. The lines and cross marks in the box plots indicate median and mean values, respectively. The ends of the boxes represent the 25th and 75th percentiles. The whiskers on the boxplot illustrate the minimum and maximum values. Asterisks indicate significant differences compared with 0% MVC (rest). Hashtags indicate differences between 5% and 25% MVC of wrist extension; *p < 0.05, **p < 0.01; # and dashed line *p* < 0.10, # and solid line *p* < 0.05.

The MEP amplitude modulation results during 5–25% MVC of wrist flexion and wrist extension are shown in Figure 3. For the ES muscle, the Friedman test showed that ES MEP amplitudes were significantly different between contraction intensities (0–25% MVC) in each task [i.e., wrist flexion and wrist extension; wrist flexion: χ^2^(5) = 14.8, *p* = 0.011; wrist extension: χ^2^(5) = 40.2, *p* < 0.001]. Specifically, *post hoc* analysis showed that the ES MEP amplitudes during ≥5% MVC of wrist flexion and wrist extension were significantly increased compared with rest (0% MVC; *p* < 0.05, Steel test; Fig. 3*A*,*D*). Moreover, the Friedman test showed no significant differences in MEP amplitudes between 5–25% MVC of wrist flexion [χ^2^(4) = 4.73, *p* = 0.316; Fig. 3*A*], while there were significant differences in MEP amplitudes between 5–25% MVC of wrist extension [χ^2^(4) = 23.3, *p* < 0.001; Fig. 3*D*]. Specially, *post hoc* analysis showed that MEP amplitudes during 15–25% MVC of wrist extension were larger compared with those during 5% MVC (*p* < 0.05; Fig. 3*D*).

For the RA muscle, the Friedman test showed no significant difference in MEP amplitudes between 0% and 25% MVC of wrist flexion and wrist extension [wrist flexion: χ^2^(5) = 9.14, *p* = 0.104; wrist extension: χ^2^(5) = 5.76, *p* = 0.330; Fig. 3*B*,*E*].

For the TA muscle, the Friedman test showed that TA MEP amplitudes were significantly different between contraction intensities (0–25% MVC) in each task (i.e., wrist flexion and wrist extension) [wrist flexion: χ^2^(5) = 21.3, *p* < 0.001; wrist extension: χ^2^(5) = 18.2, *p* < 0.01]. Specifically, *post hoc* analysis showed that the TA MEP amplitudes during ≥5% MVC of wrist flexion and wrist extension were significantly increased compared with rest (0% MVC; *p* < 0.05, Steel test; Fig. 3*C*,*F*). Moreover, the Friedman test showed no significant difference in MEP amplitudes between 5% and 25% MVC of wrist flexion and wrist extension [wrist flexion: χ^2^(4) = 4.10, *p* = 0.600 (Fig. 3*C*); wrist extension: χ^2^(4) = 3.38, *p* = 0.497 (Fig. 3*F*)].

### MEP modulation during larger contraction intensities

Since there was no significant correlation between ES background EMG activation and RA and TA MEP facilitation during 30–50% MVC of wrist flexion and wrist extension (all Spearman’s correlations *p* > 0.05), RA and TA MEP modulations during larger (30–50% MVC) contraction intensities were also compared as additional analysis.

**Figure 4.**
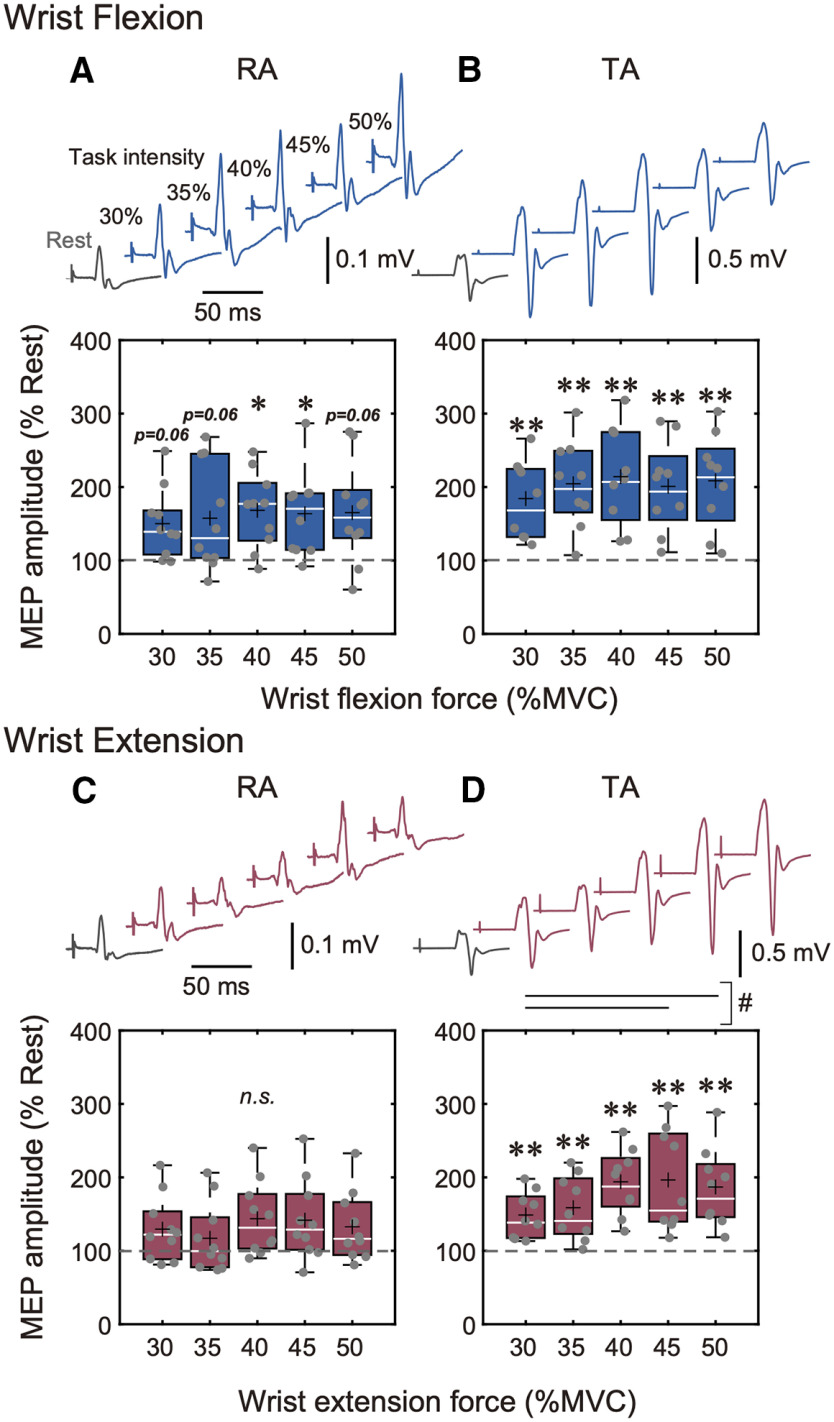
***A***, ***B***, Wrist flexion condition: averaged MEPs in the RA and TA muscles of one representative subject during 0% MVC (rest: gray traces) and 30–50% MVC (remote effect: blue traces). Box plots show group data for MEPs elicited in the RA and TA muscles. ***C***, ***D***, Wrist extension condition: averaged MEPs in the RA and TA muscles of one representative subject during 0% MVC (rest: gray traces) and 5–25% MVC (remote effect: red traces). Box plots show group data for MEPs elicited in the RA and TA muscles. All MEP amplitudes were normalized with respect to the MEP amplitude at 0% MVC (rest) for each participant. The lines and cross marks in the box plots indicate median and mean values, respectively. The ends of the boxes represent the 25th and 75th percentiles. The whiskers on the boxplot illustrate the minimum and maximum values. Asterisks indicate significant differences compared with 0% MVC (rest). Hashtags indicate significant differences between 30% and 50% MVC of wrist extension; *p < 0.05, **p < 0.01; # and solid line *p* < 0.05.

The results of the RA and TA muscle MEP amplitudes during 30–50% MVC of wrist flexion and wrist extension are shown in Figure 4. For the RA muscle, the Friedman test showed that MEP amplitudes were significantly different between rest (0% MVC) and each remote contraction condition (30%, 35%, 40%, 45%, and 50% MVC) in wrist flexion task [χ^2^(5) = 12.9, *p* = 0.0242; Fig. 4*A*], while there were no significant differences in wrist extension task [χ^2^(5) = 9.31, *p* = 0.0971; Fig. 4*C*]. Specifically, *post hoc* analysis showed that the RA MEP amplitudes during 40 and 45% MVC of wrist flexion were significantly increased compared with rest (0% MVC; *p* < 0.05, Steel test; Fig. 4*A*). The Friedman test showed no significant differences in MEP amplitudes between 30% and 50% MVC of wrist flexion [χ^2^(4) = 4.73, *p* = 0.316; Fig. 4*A*].

For the TA muscle, the Friedman test showed that MEP amplitudes were significantly different between rest (0% MVC) and each remote contraction condition (30%, 35%, 40%, 45%, and 50% MVC) in both wrist flexion and wrist extension tasks [wrist flexion: χ^2^(5) = 21.6, *p* < 0.001; wrist extension: χ^2^(5) = 31.2, *p* < 0.001; Fig. 4*B*,*D*]. Specifically, *post hoc* analysis showed that the TA MEP amplitudes during 30–50% MVC of wrist flexion and wrist extension were significantly increased compared with rest (0% MVC; *p* < 0.01, Steel test; Fig. 4*B*,*D*). The Friedman test showed no significant difference in MEP amplitudes between 30% and 50% MVC of wrist flexion [χ^2^(4) = 3.20, *p* = 0.525; Fig. 4*B*], while there were significant differences in MEP amplitudes between 30% and 50% MVC of wrist extension [χ^2^(4) = 16.6, *p* < 0.01; Fig. 4*D*]. Specially, *post hoc* analysis showed that MEP amplitudes during 45% and 50% MVC of wrist extension were larger compared with during 30% MVC (*p* < 0.05; Fig. 4*D*).

## Discussion

In the current study, we investigated whether the extent of corticospinal remote facilitation of the ES (trunk extensor) muscle induced by upper-limb contractions would depend on the task (wrist flexion or extension) and contraction intensity. Our results showed that corticospinal excitability of the ES was significantly facilitated even during low level (≥5% MVC) wrist flexion and wrist extension contractions (Fig. 3*A*,*D*). However, the extent of corticospinal remote facilitation of the ES muscle during wrist flexion did not depend on contraction intensity. On the other hand, higher levels of wrist extension contractions induced greater extent of corticospinal remote facilitation in the ES, indicating contraction intensity dependence (Fig. 3*D*). For the RA (trunk flexor) muscle, our results also showed significant corticospinal excitability facilitation during wrist flexion contractions above 25% MVC (Fig. 4*A*), but not extension (Fig. 4*C*). The extent of RA corticospinal remote facilitation was not reinforced even when wrist flexion intensity increased at 50% MVC (Fig. 4*A*). Finally, for the TA (leg) muscle, corticospinal excitability was significantly facilitated during low level (≥5% MVC) wrist flexion and wrist extension contractions (Fig. 3*C*,*F*). Specifically, the extent of corticospinal remote facilitation in the leg muscles was not changed when remote muscle contraction intensity was below 30% MVC (Fig. 3*C*,*F*), while it was increased during higher contractions (50% MVC) during wrist extension, but not flexion (Fig. 4*B*,*D*). Since there were statistically significant ES background EMG activations during wrist flexion and wrist extension at 30–50% MVC, it could be stipulated that ES background EMG activation may have affected RA and TA corticospinal remote facilitation. However, it must be noted that since the ES activation levels were exceedingly low (i.e., on average 0.8–1.9% MVC, as shown in Fig. 2*A*), and there was no statistically significant correlation between ES background EMG activation and RA and TA MEP facilitation during wrist flexion and wrist extension at 30–50% MVC (see Results, MEP modulation during larger contraction intensities), it is highly unlikely that ES background EMG activation had any considerable physiological effects on the remote facilitation in the RA and TA muscles. Therefore, it can be assumed that remote facilitation observed in the RA and TA muscles is predominantly related to wrist flexion or extension task performance. A discussion about possible mechanisms of task- and intensity-dependent corticospinal remote facilitation follows.

### Task and intensity dependence of arm-trunk corticospinal remote facilitation

The main findings of our current study are that: (1) corticospinal remote facilitation of the ES muscle (trunk extensor) was elicited even during low-level wrist flexion and extension contractions (≥5% MVC), while relatively strong wrist flexion and extension contractions (≥25% MVC) were required to induce remote facilitation of the RA muscle (trunk flexor); and (2) extent of corticospinal remote facilitation of the ES (trunk extensor) was proportional to the contraction intensity of wrist extension but not wrist flexion, while this was not observed for the RA (trunk flexor) muscle during both wrist extension and flexion tasks. Moreover, corticospinal excitability in the TA muscles was significantly facilitated during low level (≥5% MVC) wrist extension and wrist flexion contractions. The extent of corticospinal remote facilitation in the leg muscles was not changed when remote muscle contraction intensity was below 30% MVC, while the extent of corticospinal remote facilitation increased during higher wrist extension contractions (50% MVC), but not flexion. It has previously been reported that corticospinal remote facilitation between upper-limb and lower-limb muscles (interlimb facilitation) was achieved regardless of the task that was performed (Chiou et al., 2013a,b; Tazoe and Komiyama, 2014). Moreover, the extent of interlimb corticospinal remote facilitation was shown to depend on voluntary effort level of the contracted muscle (Kawakita et al., 1991; Tazoe et al., 2007b, 2009; Tazoe and Komiyama, 2014). However, it was unclear until now if these flexion/extension task characteristics of interlimb neural interactions would remain similar during arm-trunk interactions. Based on previous studies showing that trunk flexor and extensor muscle activity depends on the direction of the arm movements (Aruin and Latash, 1995; Hodges et al., 1997), we hypothesized that the extent of arm-trunk corticospinal remote facilitation would depend on the task performed during upper-limb muscle contractions (i.e., flexion or extension). We also hypothesized that muscle contraction intensity would affect the extent of arm-trunk remote effect facilitation, similar to that of interlimb facilitation (Tazoe et al., 2007b, 2009; Tazoe and Komiyama, 2014). Consistent with these hypotheses, our results demonstrated that the extent of corticospinal remote facilitation between arm and trunk muscles depended on the task (i.e., upper-limb flexion or extension) and the level of remote upper-limb muscle contractions. A study by Chiou et al. (2016) previously reported that corticospinal excitability of the ES muscle was greater during a rapid shoulder flexion task (phasic contractions) compared with a static shoulder flexion task (tonic contractions), while the extent of remote facilitation of the RA muscle was similar between these tasks. Our results also showed that the profiles of remote facilitation during deferent level of contraction differed between tasks (i.e., wrist flexion and extension). Therefore, ES muscle corticospinal facilitation was affected to a different extent depending on the task during arm movements, while this was not true for the RA muscle. Specifically, our study showed that ES muscles could be facilitated more when wrist extensor (but not flexor) contraction intensities were increased, even if the effort changes were exceedingly small (i.e., 5% MVC). This may suggest that wrist extensors have a stronger connectivity with the trunk extensors (ES) compared with the trunk flexors (RA). Such functional muscle connectivity (extensor-extensor/flexor-flexor connectivity) is also supported by our results that remote facilitation of trunk flexors (RA) occurred during contractions of wrist flexors as well as that contraction intensity dependent remote facilitation of the leg extensors (TA) was observed during contractions of the wrist extensors.

On the other hand, it has previously been reported that ES corticospinal excitability was facilitated to a larger extent by elbow flexion compared with elbow extension, which suggests that elbow flexors have the stronger interactions with trunk extensors (Chiou et al., 2018). Therefore, it is also possible that arm and trunk connectivity may not always depend on the flexor/extensor remote muscle pairs, but also on the functional connectivity between these muscles. In our current study, ES MEPs were facilitated during both wrist flexion and extension at contraction intensities above 5% of MVC effort, although contraction intensity dependence was only shown during wrist extension. Therefore, ES muscles could detect arm movements even at relatively small contraction intensities regardless of whether wrist extension or flexion was performed. It has been reported that activation of ES muscles has an important role for minimizing postural displacement during arm movement-induced postural perturbations (Aruin and Latash, 1995; Hodges et al., 1997). Taken together, such functional connectivity of the ES and remote limb muscle pairs may perhaps explain greater sensitivity of trunk extensors to upper-limb movement and changes in corticospinal excitability to contraction intensity, as demonstrated in our current study.

### Possible mechanisms of arm-trunk corticospinal remote facilitation

Since it is well known that the excitability of the corticospinal pathway is affected by excitation of both cortical and spinal circuits (Hess et al., 1987); arm-trunk corticospinal remote facilitation, which was demonstrated in our current study, could also be attributed to cortical and/or spinal circuits. Indeed, previous studies reported that inter-limb remote facilitation could affect both cortical (Tazoe et al., 2007a; Chiou et al., 2013a,b) and spinal (Jendrássik, 1883; Kawamura and Watanabe, 1975; Borroni et al., 2005) motor circuits. Specifically, it was demonstrated that remote limb muscle contractions decreased upper-limb and lower-limb muscle short-interval intracortical inhibition (induced by paired-pulse TMS), which implies cortical inhibition mechanisms during remote muscle contractions (Chiou et al., 2013a,b). Similarly, decreased duration of the cortical silent period (induced by TMS during low levels of muscle contractions) of upper-limb muscles was shown to be elicited by contractions of lower-limb muscle, also suggesting cortical inhibition (Tazoe et al., 2007a). These studies suggest that cortical disinhibition may contribute to corticospinal remote facilitation between upper-limb and lower-limb muscles. Moreover, H-reflex responses elicited by peripheral nerve stimulation (Jendrássik, 1883; Kawamura and Watanabe, 1975; Borroni et al., 2005) as well as posterior-root spinal reflex responses elicited by transcutaneous spinal cord stimulation (Kato et al., 2019; Masugi et al., 2019; Sasaki et al., 2020a) in the upper-limb or lower-limb muscles were facilitated by remote limb muscle contractions. These studies indicate spinal reflex remote modulation mechanisms also contribute to interlimb remote facilitation. Therefore, arm-leg remote facilitation observed in our current study (i.e., TA remote facilitation during wrist flexion and wrist extension) may also be caused by cortical and/or spinal mechanisms. Taken together, it may be speculated that arm-trunk corticospinal remote facilitation is also mediated in cortical and/or spinal networks. Conversely, two recent studies suggested that cortical-levels networks may primarily be attributed to arm contraction-induced trunk remote facilitation (Chiou et al., 2018; Sasaki et al., 2020b). Specifically, Chiou et al. (2018) showed decreased short-interval intracortical inhibition, indicating disinhibition of intracortical circuits. However, no changes in the cervicomedurally MEPs (induced by cervicomedullary junction magnetic stimulation) of the trunk muscle during upper-limb contractions were observed, suggesting that subcortical (spinal) excitability was unaffected (Chiou et al., 2018). Similarly, it was shown that cervicomedurally MEPs of the trunk muscles were not affected by upper-limb contraction, while corticospinal excitability was modulated (Sasaki et al., 2020b). Although it is still possible that both cortical and/or spinal networks may be involved, recent evidence suggests that arm-trunk remote facilitation is more likely mediated in the cortical-level networks. The cortical remote facilitation mechanisms hypothesis is also supported by basic animal studies which have demonstrated that intracortical facilitation may be involved in spreading of neural activity within the motor cortex (Capaday et al., 2009, 2011). Specifically, it was shown that neural activity initiated at a cortical locus can spread to the neighboring cortical regions which represent different muscles via intrinsic horizontal connections between neurons within the motor cortex (Capaday et al., 2009, 2011). In human studies using TMS, it was proposed that similar corticocortical connections may exist (Boroojerdi et al., 2000; Komeilipoor et al., 2017). Specifically, previous studies investigating corticospinal remote facilitation mechanisms proposed that activation of cortical motor networks by voluntary contraction of certain muscles could spread to neighboring cortical areas representing different segment muscle (Boroojerdi et al., 2000) and that the extent of this spreading may depend on the distance between M1 representations of different muscles within the cortex (Boroojerdi et al., 2000; Sasaki et al., 2018). This suggests that cortical remote facilitation mechanisms are dependent on the anatomic somatotopic representations within the M1. Moreover, it is well known that somatotopy of muscles in the different body segments overlap within the motor cortex (Penfield and Boldrey, 1937; Brasil-Neto et al., 1992). Since the trunk muscle representations in the homunculus of the M1 in humans is located near the upper-limb representations, it is likely that activation of trunk cortical motor circuits was also induced during voluntary contraction of upper limbs because of the overlapping of the cortical representations within the M1. Therefore, arm-trunk corticospinal remote facilitations observed in our current study, are likely modulated via anatomic connections such as intracortical connectivity networks, and/or overlapping of somatotopic representations at the supraspinal level, although subcortical mechanisms cannot be fully ruled out. Indeed, previous studies reported that subcortical circuits may be more involved as contraction levels increase (Stedman et al., 1998; Muellbacher et al., 2000). Therefore, it is possible that subcortical mechanisms may also have contributed to the remote facilitation when contraction levels were higher. Moreover, the proximity of motor representations within M1 between remote muscles may be one of the possible mechanisms related to the corticospinal remote facilitation (Boroojerdi et al., 2000). However, since our current study showed that profiles of remote effect of ES and RA muscles were different, despite their proximity within M1, remote facilitation mechanisms cannot only be explained by somatotopic relationships. A specific discussion related to other possible mechanisms of corticospinal remote facilitation follows below (Significance of corticospinal remote facilitation).

### Significance of corticospinal remote facilitation

Overall, corticospinal remote facilitation relationship between certain remote muscles (e.g., arm-trunk or arm-leg) may reflect anatomic relationships with the central nervous system and/or functional connectivity between these muscles. If only the anatomic relationship between remote muscles (i.e., proximity of motor representations within M1; as discussed above, see Possible mechanisms of arm-trunk corticospinal remote facilitation) were to determine the profile of remote corticospinal facilitation, remote effect between arm-trunk would simply be effective compared with that of arm-leg. However, our current results showed that even different trunk muscles (i.e., ES and RA), which are located very close with the M1 (Tsao et al., 2011), had very distinct remote facilitation responses. Moreover, the leg muscles (TA) showed lower threshold of remote facilitation, compared with the trunk flexor muscles (RA). Therefore, our current study may suggest that functional relationships between remote muscles are also represented within the corticospinal circuits, in addition to their anatomic relationship (i.e., somatotopic representations with M1). Specifically, ES muscles have an important functional role for maintaining postural stability during arm movements (Aruin and Latash, 1995; Hodges et al., 1997), which may be why ES muscle showed lower thresholds and task- and intensity-dependent modulation of corticospinal remote facilitation. On the other hand, interlimb (arm-leg) coordinated movements are functionally relevant during rhythmic movements which activate the central pattern generator (CPG), such as walking and cycling (Zehr and Duysens, 2004). Indeed, it was shown that arm-leg neural interaction could be strengthened to a larger extent during rhythmic movements, compared with tonic contractions (Frigon et al., 2004; Zehr et al., 2007). Therefore, under the tonic contractions condition in the current study, intensity-dependent changes in arm-leg corticospinal remote facilitation may not be functionally required. However, intensity-dependent changes in arm-leg corticospinal remote facilitation were observed only during high levels of wrist extension intensities in the current study. It was reported that rhythmic ipsilateral hand and foot movements performed at the same time are made more reliable when they are synchronized in the same direction (Baldissera et al., 1982). A previous study investigating effects of rhythmic ankle plantar/dorsi flexion on H-reflex excitability of wrist flexors (i.e., flexor carpi radialis) also reported that modulation peak of H-reflex in the wrist flexors occurred at the same time as the contraction of the ankle plantar flexors (i.e., soleus muscle; Baldissera et al, 2002). Moreover, preference of rhythmic hand and foot movements reflects spatial rather than structural constraints. When the hand was pronated, wrist flexor neural pathways were facilitated during the plantarflexion phase, while when the hand was supinated, wrist extensors were facilitated during the plantarflexion phase, and wrist flexors were facilitated during dorsiflexion (Borroni et al., 2004). Therefore, rhythmic movements eliciting CPG-like activations were shown to strengthen arm-leg connectivity depending on the direction of the movement, rather than based on specific muscle pairs, which indicates that rhythmic interlimb interactions may reflect functional connectivity (Borroni et al., 2004). On the other hand, tonic contraction tasks, which were performed in our current study, strengthened the connectivity in specific muscle pairs (i.e., ECR and TA) during high levels of contraction. This may possibly reflect a biological (structural) characteristic, rather than a functional connection. Moreover, trunk flexors (RA) have an anatomic advantage in that the representation within the homunculus of the motor cortex is located close to the upper-limb representations. Despite this, the RA showed higher threshold of remote facilitation and no task- and intensity-dependent modulation. This may reflect weak functional connectivity between trunk flexors (RA) and arm muscles. Moreover, different neural innervations of these muscles may also contribute to their remote facilitation profiles. Specifically, the ES muscles at the T12 level are innervated by dorsal rami of thoracic and lumbar spinal nerves (T8–L3), while the RA muscles are innervated by the intercostal nerve (Pradhan and Taly, 1989). Therefore, it is possible that the neural innervations may also have contributed to remote facilitation profiles in our study. Overall, multiple effect including anatomic somatotopic relationships as well as functional connectivity may attribute to task-dependent and intensity- modulation of remote facilitation between arm and trunk extensor. Our findings therefore inform a more comprehensive understanding of the basic principles of human motor control related to the arm-trunk neural interaction. Taken together, these results may also suggest that functional movement synergy oriented training is crucial in rehabilitation to strengthen arm-trunk interactions as a way for improving functional performance after neurologic impairments such as spinal cord injury (Chiou and Strutton, 2020; Chiou et al., 2020).

### Limitations

Our work has several limitations that should be noted. First, some previous studies investigating interactions between trunk and upper-limb muscles have examined contralateral side trunk muscles to the contracted arm to elicit MEP responses (Davey et al., 2002; Chiou et al., 2018; Chiou and Strutton, 2020), while we chose to investigate the ipsilateral side in the current study. Davey et al. (2002) previously reported that ES muscle activity in the contralateral side to contracted arm was increased when arm muscle contractions during shoulder abduction were increased, while that of ipsilateral side was not affected considerably. Therefore, ipsilateral side was investigated in our study with the aim to minimize co-contraction of ES muscles during wrist flexion and extension, although it is also likely that ES background EMG activations in the contralateral and ipsilateral side muscles were similar in sitting posture (Sasaki et al., 2020b). Nonetheless, further work is warranted to systematically examine differences in remote effect facilitation between ipsilateral and contralateral side trunk muscles during various tasks at intensities.

Second, although significant ES background EMG activations during strong wrist flexion and extension contractions (>25% MVC) were exceedingly low (i.e., on average 0.8–1.9% MVC, as shown in Fig. 2*A*), and there were no significant correlations between the remote RA and TA MEPs and ES background EMG activations during 30–50% MVC of wrist flexion and wrist extension, we still cannot completely exclude a possibility that these slight ES activations could have affected the profiles of remote effect facilitation of RA and TA muscles, in additions to wrist flexion and extension contractions.

## Synthesis

Reviewing Editor: Mark Laubach, American University Decisions are customarily a result of the Reviewing Editor and the peer reviewers coming together and discussing their recommendations until a consensus is reached. When revisions are invited, a fact-based synthesis statement explaining their decision and outlining what is needed to prepare a revision will be listed below. The following reviewer(s) agreed to reveal their identity: Paul Strutton.

Your manuscript was reviewed by two experts in your field and they both felt that substantial revisions are needed before the manuscript can be considered for publication. Please respond to all points raised in the reviews shown below in revising your manuscript. Thank you.

Reviewer 1

The purpose of the current study was to examine the neural interactions between the arm and the trunk and between the arm and the leg by probing corticospinal excitability changes during wrist contractions. Twelve healthy male subjects undertook contractions of the dominant wrist (flexion and extension to varying levels) during which motor evoked potentials (MEPs) to transcranial magnetic stimulation were recorded from trunk and leg muscles (erector spinae [ES], rectus abdominis [RA] and tibialis anterior [TA]). Results showed that MEPs were facilitated in ES during low level wrist flexion and extension, but higher levels of contractions were required to facilitate MEPs in RA. Further, the facilitation of ES MEPs during wrist extension depended on the level of contraction, this was not the case for wrist flexion. Finally, MEPs in TA were facilitated during low level wrist contractions, but dependency on contraction strength was only observed in stronger wrist extension. The authors conclude that there is task and intensity dependency on corticospinal excitability to ES muscles which is not observed for RA or TA muscles and suggest that these results may reflect function of the muscles studied.

Overall, the paper has been very well written, experiments appear to have been performed diligently and presentation of results and the figures are very good. I have some questions regarding the site chosen for the recording of the MEPs and suggestions for the discussion.

Major comments:

Previous work on the interactions between the trunk and upper limb movements have often used contractions of one upper limb and found corticospinal excitability changes on the opposite side (e.g. Davey et al., 2002; Chiou et al 2018; Chiou & Strutton 2020). Why was the same side chosen for the contraction and the recording of MEPs, since when a stimulus is delivered to the left primary motor cortex, MEPs will be generated in the contracting wrist flexors/extensors?

I feel that there should be some discussion on the crossed facilitation effect being related to the level of background contraction of the remote muscle in addition to the already included discussion (in section 4.2). As contraction levels increase, there is evidence of subcortical/spinal mechanisms may be more involved in the effect (see Muellbacher et al., 2000 and Stedman et al. 1998). There is also not much discussion of the possible mechanisms of the effects seen in the TA MEPs during wrist flexion/extension, only to state that under tonic conditions, interactions between the arm and leg may not be functional required. But MEPs in TA were increased in higher levels of wrist extension. Could the authors speculate on the mechanisms of this?

Minor comments:

The stimulus intensity was set at 120% of the motor threshold (MT) level for ES. However, were there any subjects where the MT was above ∼83 and 120% MT could not be used?

Page 5, line 14 - “During the experiment, participants were seated comfortably on a chair.” I presume that subjects had back support, the level of EMG in ES (figure 2) is very low if not?

Page 5, line 8 & 9. I would suggest providing a little more information regarding exclusion criteria for TMS studies, e.g. those listed in (Rossi et al. 2011).

Page 7, line 4-6 “The average MEP amplitudes with standard errors at rest in the RA, and TA muscles were 0.34 {plus minus} 0.06 mV and 0.42 {plus minus} 0.06 mV, respectively.” Why use SEM in this particular sentence, when earlier SD is used, e.g. P5, line 7.Also, why are the ES MEPs amplitudes not also reported here for comparison?

Page 7, lines 11-13. I think it would be better to use the original papers, rather than a review, to give credit to the researchers who initially report these findings on effect of background EMG on MEP size (Hess et al.,1987)

Page 7, line 26 - “consistent to” should be “consistent with"

Figure 1B - spelling - “rondomized"

Figure 3 - legend states “Hashtags indicate differences between 5-25% MVC of wrist flexion and wrist extension.” But there are no hashtags above the flexion graphs, are they missing or is this a typo? Also, what is the purpose of reporting P<0.1?

Figures 3&4 - are the representative MEPs averaged, or individual MEPs?

References cited in this review

Chiou, SY, Strutton, PH, Perez MA. (2018). Crossed corticospinal facilitation between arm and trunk muscles in humans. J Neurophysiol. doi: 10.1152/jn.00178.2018.

Chiou SY, Strutton PH. Crossed Corticospinal Facilitation Between Arm and Trunk Muscles Correlates With Trunk Control After Spinal Cord Injury. (2020). Front Hum Neurosci. Oct 23;14:583579. doi: 10.3389/fnhum.2020.583579.

Davey NJ, Lisle RM, Loxton-Edwards B, Nowicky AV, McGregor AH. (2002). Activation of back muscles during voluntary abduction of the contralateral arm in humans. Spine 27: 1355-1360.

Hess, C. W., Mills, K. R. & Murray, N. M. F. (1987). Responses in small hand muscles from magnetic stimulation of the human brain. Journal of Physiology 388, 397419.

Muellbacher W, Facchini S, Boroojerdi B, Hallett M. (2000). Changes in motor cortex excitability during ipsilateral hand muscle activation in humans. Clin Neurophysiol 111: 344-349. doi:10.1016/S1388-2457(99)00243-6.

Rossi S, Hallett M, Rossini PM & Pascual‐Leone A (2011). Screening questionnaire before TMS: an update. Clin Neurophysiol 122, 1686.

Stedman A, Davey NJ, Ellaway PH. (1998). Facilitation of human first dorsal interosseous muscle responses to transcranial magnetic stimulation during voluntary contraction of the contralateral homonymous muscle. Muscle Nerve 21: 1033-1039. doi:10.1002/(SICI)1097-4598(199808)21:8<1033::AID-MUS7>3.0.CO;2-9.

Reviewer 2

The authors examined remote effect facilitation in trunk and leg muscles during voluntary isometric contractions of wrist muscles. They found that the remote facilitation in the erector spinae (ES) muscles was different from that in the rectus abdominis (RA) muscles and influenced by levels of the contractions of the wrist muscles. They suggested the remote facilitation observed in the study was due to activation of the wrist motor representations spreading to the trunk motor representations. Findings are interesting and the results look convincing. However, I have concerns on including data of RA and tibalis anterior (RA) at higher levels of contractions (>30%) while there was an increase in activity in the ES muscles. I am also not convinced by the proposed mechanisms of anatomical connections (page 16, line 3-4) contributing to the different facilitation observed between the ES and RA during voluntary contractions of the wrist. Finally, the rationale for conducting this study was not clear to me in the Introduction.

1. The authors reported increases in activity in the ES muscles when the wrist flexors or extensors were contracting more than 25% of maximal voluntary contractions (MVCs). Given that a small amount of voluntary contractions of a muscle is sufficient to increase corticospinal excitability of a resting muscle (i.e. 5%), it is difficult to know whether it was the contractions of the wrist muscles or the increased activity in the ES muscle that contributed to the remote effect facilitation in the RA muscles at the higher level of contractions. Similarly, the remote facilitation observed in the tibalis anterior (TA) muscles at the higher level of contractions is also difficult to interpret as in what was the cause of increases in MEPs in the TA muscle. Although background EMG in the ES muscle did not correlate with amplitudes of MEPs in RA or TA, there is no evidence showing muscle activity correlating with the amount of remote effect facilitation. Therefore, a non-significant correlation result is not able to rule out the potential influence of activity in the ES muscle on the corticospinal excitability of the RA or TA. I think this part of the results should be removed from the manuscript. Discussion and conclusion will also need to be modified if this part of the results are removed.

2. The RA motor representations are close to the ES motor representations and yet the results obtained from these two muscles are different. If the remote facilitation was due to the activation in the wrist motor representations spreading across to the trunk and leg motor representations, it would be expected that the facilitation in the RA would be similar to the ES. However, the authors found no facilitation in the RA at 25% of MVC of wrist flexors/extensors. The potential mechanisms in the Discussion need to be reconsidered and revised. I also found the discussion around different neurophysiological behaviours between the ES and RA weak. I think this finding is probably the most novel part of this study and needs to be interpreted and discussed properly. For example, could the different results found between the ES and RA be because the neural innervations to the two muscles are different?

3. The authors suggest the mechanisms underlying the remote effect facilitation are modulated by cortical mechanisms. This might be true when the level of contractions is low. Previous studies (e.g. Perez and Cohen 2008) showed that subcortical circuits also contribute to the remote facilitation when the level contraction is higher. Given that the authors included various levels of contractions, the subcortical contribution to the remote facilitation should be discussed.

4. The authors quotes several previous articles related to remote facilitation between limbs as well as between arms and the trunk. According to those studies, the remote facilitation is task-dependent and force-dependent regardless whether it was a limb or a trunk muscle being studied. We also know that contractions of the arms facilitate corticospinal excitability of the trunk and the effects are task-dependent. Hence, the current findings are largely expected, damaging the novelty of the work. While the findings in the RA were interesting, the rationale for measuring this muscle was not explained. What is the evidence leading the authors to think that the RA may behave differently from the ES in the remote facilitation? Was it expected that wrist flexors and extensors induced different remote facilitation in the ES and RA? What was the purpose of including a leg muscle in the study? The study was not supported by a clear hypothesis and that may explain the incomplete data interpretation in the Discussion.

5. Page 8, line 18-27, Statistical analysis. It was unclear to me what were the parameters included in the first and the second Friedman tests. Please clarify the parameters or revise the paragraph.

6. Page 10, line 16-18. It was unclear to me whether amplitudes of MEPs in the ES muscle were similar between 10%, 15%, 20%, and 25% but greater than at 5%. There is a multiple comparison issue here. Was the p-value adjusted to account for the multiple comparisons?

7. Page 13, line 11-12. The authors claimed the study as the first one showing task-dependency of the remote effect facilitation between the arm and trunk muscles. However, in the next paragraph they cited a previous study (Chiou et al., 2018) reporting the remote effect facilitation in the trunk extensors during elbow flexors and extensors. Hence, the claim is untrue as this is not the first study showing the task-dependent effect of the remote facilitation between the arm and the trunk.
